# Implications of movement-related cortical potential for understanding neural adaptations in muscle strength tasks

**DOI:** 10.1186/1755-7682-7-9

**Published:** 2014-03-06

**Authors:** Eduardo Lattari, Oscar Arias-Carrión, Renato Sobral Monteiro-Junior, Eduardo Matta Mello Portugal, Flávia Paes, Manuel Menéndez-González, Adriana Cardoso Silva, Antonio Egidio Nardi, Sergio Machado

**Affiliations:** 1 Laboratory of Panic and Respiration, Institute of Psychiatry, Federal University of Rio de Janeiro, Rio de Janeiro, RJ, Brazil; 2 Unidad de Trastornos del Movimiento y Sueño (TMS), Hospital General Dr. Manuel Gea Gonzalez, Secretaria de Salud, México, DF, Mexico; 3 Brazilian Institute of Rehabilitation Medicine, IBMR, Rio de Janeiro, Brazil; 4 Neuroscience of Exercise Laboratory, LaNEx, Iguaçu University, Rio de Janeiro, Brazil; 5 Neuroscience of Exercise Laboratory, Institute of Psychiatry, Federal University of Rio de Janeiro (IPUB/UFRJ), Rio de Janeiro, RJ, Brazil; 6 Institute of Phylosophy (IFILO), Federal University of Uberlândia (UFU), Minas Gerais, Brazil; 7 Physical Activity Neuroscience, Physical Activity Postgraduate Program, Salgado de Oliveira University (UNIVERSO), Niterói, RJ, Brazil; 8 Quiropraxia Program of Faculty of Health Sciences, Central University (UCEN), Santiago, Chile; 9 Neurology Unit, Hospital Álvarez-Buylla, Mieres, Spain

**Keywords:** Movement-related cortical potential, Acute effects, Chronic effects, Counter resistance training

## Abstract

This systematic review aims to provide information about the implications of the movement-related cortical potential (MRCP) in acute and chronic responses to the counter resistance training. The structuring of the methods of this study followed the proposals of the PRISMA (*Preferred Reporting Items for Systematic reviews and Meta-Analyses).* It was performed an electronically search in Pubmed/Medline and ISI Web of Knowledge data bases, from 1987 to 2013, besides the manual search in the selected references. The following terms were used: *Bereitschaftspotential*, *MRCP*, *strength and force*. The logical operator “AND” was used to combine descriptors and terms used to search publications. At the end, 11 studies attended all the eligibility criteria and the results demonstrated that the behavior of MRCP is altered because of different factors such as: force level, rate of force development, fatigue induced by exercise, and the specific phase of muscular action, leading to an increase in the amplitude in eccentric actions compared to concentric actions, in acute effects. The long-term adaptations demonstrated that the counter resistance training provokes an attenuation in the amplitude in areas related to the movement, which may be caused by neural adaptation occurred in the motor cortex.

## Introduction

Traditionally, strength gains acquired during the early stages of a counter-resistance training program, mainly in untrained individuals, are explained by a neural adaptation without noticeable hypertrophy [1,2]. There are several lines of evidence supporting this neural adaptation related to counter-resistance training, such as an increase in amplitude of electromyographic activity (EMG), a transient increase in firing rate of motor units, synchronization of motor units, use of imagined contractions (mental practice), cross education and reduction in antagonist co-activation [1]. The main neural adaptations that take place through the practice of counter-resistance training occur at two levels: (a) supraspinal level, involving changes in excitability and corticospinal inhibition, and (b) spinal cord level (spinal cord), involving changes in spinal motoneurons and inhibitory and excitatory interneurons [2,3]. Among the techniques used to measure the neural adaptations occurred due to counter-resistance training, changes in electromyographic activity (EMG) are observed in association with activation patterns between agonists, antagonists and synergics muscles [3,4]. The majority of findings above demonstrates adaptations occurring mainly at the spinal level, with little neurophysiological evidences supporting the paradigm of adaptation in the cerebral cortex. The technical investigation limitations of the activity of primary motor cortex in a human being reduce the number of evidences that support the initial strength gains related to changes in supraspinal level [3]. Studies involving the adjustments that take place in the motor cortex with counter-resistance training were performed through magnetic transcranial stimulation (TMS) [5,6] and through movement related-cortical potentials (MRCP) [7-12]. These studies point to increases in muscular strength accompanied by an increase in amplitude of evoked potential motor [6] and as an anticipatory stage of the movement in tasks involving muscle strength, respectively.

When an individual performs a volitional movement (i.e., self-paced), brain waves can be observed in ongoing electroencephalographic (EEG) activity, preceding the movement and possibly are an expression of the brain preparation to execute the desire movement. This distinguished signal related to volitional movement is called MRCP. It reflects an anticipatory mechanism that is crucial to motor skill performance [13]. In muscle strength tasks, this anticipatory mechanism has been usually observed [14]. The existence of this negative slow wave before the voluntary movement of one second or more was first reported more than 40 years ago [15]. The rationale that MRCP is related to motor planning phase in a task force is due to the fact that they start about 1.5 s before the movement generated by neural circuits is verified by electroencephalographic records. The first phase of MRCP is characterized by a negative potential (upward deflection) which begins 600–1200 ms before the muscle activity, named *Bereitschaftspotential*[16] or “Readiness Potential” (RP). Another study suggests that this first stage begins nearly 2 s before initiating the movement in the supplementary pre-motor area (pre-SMA) nonspecifically regarding the site and in the adequate supplementary motor area (SMA) according to the somatotopical organization, and right after the lateral premotor cortex [17]. The second phase consists of a period after RP, it occurs nearly 400 ms before the muscular contraction, the slope of this negative potential becomes more gradual and occurs at the contralateral primary motor cortex and lateral premotor cortex [17]. This phase is named negative slope (NS) [18,19]. These two phases are followed by the motor potential (MP), which can be observed before and after the movement [19]. The motor potential precedes the negative slope stage and comprises a period of nearly 150 ms [19].

Studies related to MRCP and muscle strength showed supplementary motor area, sensorimotor cortex and premotor cortex are the main contributor areas to a high amplitude of MRCP [8,10]. Data indicates that the discharges of pyramidal tract cells necessary to produce a particular movement are generated in these areas, and reach the peak particularly in the contralateral cortex [10]. However, animal studies have shown that there are cell populations that only discharge in phases before a motor task and others that only tonically discharge during the task exploration phase [20]. Thus, MRCP cannot be used during sustained contractions, but it is an interesting tool, especially for studying central alterations during repetitive contractions [9].

There are not many studies in the literature that demonstrate the changes in central levels, by the plasticity displayed by various supraspinal centers after counter-resistance training referred by MRCP. This paper aims to review the behavior of MRCP in tasks of muscle strength of acute and chronic forms, increasing the understanding of neural adaptations after counter-resistance training.

## Methods

### Eligibility criteria

The structuring of the methods of this study will follow the proposals of the PRISMA (*Preferred Reporting Items for Systematic reviews and Meta-Analyses*) [21]. Thus, the approach will be adopted PICOS (population, intervention, group being compared, results and research design), for the determination of eligibility.

1-Type of study- RCTs and non-randomized studies that evaluated the behavior of the MRCP tasks in muscle strength in both the acute and chronic effects;

2-Types of sample- Subjects were all right-handed, with no history of pathologies to either the hand or wrist. All individuals were healthy and had no known neuromuscular disorders, musculoskeletal injury or neurological events;

3-Types of intervention- undergoing a task force exercise being conducted unilaterally by comparing the acute and chronic effects between the proposed interventions on the behavior of MRCP;

4-Types of measures- Measures of the electroencephalogram (EEG) are analyzed by Bereitschaftspotential also referred to as Readiness Potential (RP), Negative Slope (NS) and Motor Potential (MP).

### Sources of information

The methodological design of the present study consisted of the search and analysis of English articles that investigate the MRCP behavior in tasks of muscle strength, in acute and chronic forms. A search was performed in the databases Pubmed/Medline and ISI Web of Knowledge, published until the month of June 2013. Experts on the subject of the present study were also contacted to send articles. To find additional articles, all tables were examined for evidence of previous systematic reviews and found references to randomized controlled trials and controlled as necessary. In addition, we analyzed the references of all selected articles. Searches were closed on 10 June 2013.

### Search

The following terms were used: *Bereitschaftspotential*, *MRCP*, *strength and force*. The logical operator “AND” was used to combine descriptors and terms used to search publications.

### Selection of studies

The selection of studies was performed by two independent evaluators, that in case of disagreement sought a consensus on the selection. The evaluation consisted of the filtration studies, from the analysis of the title, followed by an analysis of the summary and then the full article. The need to settle possible disagreements between the two reviewers, a third reviewer was requested due to the end. Complete relevant articles were obtained and assessed eligibility and exclusion.

### Data collection

The following data were extracted from the articles: sample size, participant characteristics, muscles used, setting strength exercise, assign the experiment, the main results related to the behavior of MRCP and electrodes used. In addition, several other information about the methods and outcomes were collected. These procedures were performed by two independent investigators, who reached a consensus in case of disagreement.

### Exclusion criteria

We excluded articles that had no effective intervention strength exercise, those using the other intervention associated with physical exercise that could create a risk of bias in the study, composite samples of children, adolescents, the elderly, individuals with mental illness or neurological, neuromuscular disorders, musculoskeletal injuries, those who do not have detailed statistical procedure applied, or not presented the results of specific measures related EEG MRCP.

## Results

Based on the defined criteria, 97 articles were found in the search conducted in the literature. After an initial screening, 41 articles were excluded, after duplicates removed. Subsequently, 36 articles were excluded based on the title or abstracts that did not meet the criteria adopted in the study. Of the 20 remaining articles, three were excluded by the characteristics of the sample (n = 3) and three for other types of intervention (n = 3).

With the terms used in this work, fourteen studies were selected which were properly met the criteria for this review. Table 1 shows a summary of them.

**Table 1:** Summary of MRCP studies for acute and chronic effect on strength

**Author (year)**	**Sample**	**Muscle(s)**	**Strength exercise protocol**	**Experimental design**	**MRCP Behavior**	**Electrodes used**
Freude et al. [22]		Flexor digitorum superficialis, flexor digitorum profundus	20%, 50% and 80% of MVIC in the handgrip, with and without intentional fatigue	Acute	The highest strength levels were well correlated with RP. With 80% of CVM in fatiguing situation and 20% without fatigue, there have been increases in RP, which did not occur with 50% of CVM.	C3, Cz, C4
Oda and Moritani [8]	11 right-handed men	First dorsal interosseous, flexor digitorum profundus, flexor digitorum superficialis	50% and 10% MVIC handgrip	Acute	Increase in amplitude of negative slope (NS) in Fz, with 50% CVM > 10% CVM.	C3 and C4
Shibata et al. [10]	10 right-handed men	Biceps brachialis	3 tasks: 1^st^ - autodynamic “shots” with 20% of MVIC; 2^nd^ - keep 2 seconds with 20% of MVIC; 3^rd^ - the same as the 2^nd^ task with arterial occlusion	Acute	The mean amplitude of MRCP was higher in tasks 2 and 3 and in the respective electrodes	C3, Cz, C4
Siemionow et al. [12]	6 men and 2 women, both right-handers	Biceps brachialis and brachioradialis	10, 35, 60 and 85% of elbow flexion MVIC; 3 Rates of strength development (slow, moderate and fast) with 30% MVIC of elbow flexion	Acute	High correlation between MRCP and strength levels with r = 0.84 (SMA) and r = 0.85 (sensory-motor).	C3, Cz, C4
Fang et al. [25]	6 men and 2 women, both right-handers	Biceps brachialis, brachioradialis, triceps brachialis and deltoid	50 eccentric voluntary contractions and 50 concentric voluntary contractions with 10% load of the body weight	Acute	Increase in amplitude of the negative slope (NS), being higher for eccentric action than concentric action	C3, Cz, C4 and Fz
Siemionow et al. [11]	8 patients with chronic fatigue syndrome (SFC) (5 men and 3 women) and 8 healthy individuals (5 men and 3 women)	First dorsal interosseous, flexor digitorum profundus, flexor digitorum superficialis and finger extensors	50% of MVIC handgrip in two situations: fatiguing task (FT) and non-fatiguing task (NFT)	Acute	Increase in amplitude of the negative slope (NS) the SFC group was significantly higher than the control group, both in FT and NFT. In the SFC group, the amplitude of NS was higher in FT than in NFT.	C3, Cz, C4
Fang et al. [24]	6 men and 2 women, both right-handers	Biceps brachialis, brachioradialis, triceps brachialis and deltoid	40 maximal eccentric voluntary contractions and 40 maximal concentric voluntary contractions, both in an isokinetic dynamometer	Acute	Increase in negative slope in the eccentric compared to concentric phase	C3, C4, C6, F4, FC4 and FC6
Liu et al. [23]	8 men and 1 woman	Flexor digitorum superficialis, flexor digitorum profundus and biceps brachialis	200 intermittent MVIC on handgrip	Acute	Without significant difference	C3, Cz, C4, Fz and Pz
Do Nascimento et al. [7]	14 men and 1 woman, both right-handers	Soleus and anterior tibial	Plantar flexion, isometric plantar flexion (real or imaginary) in two different rates of force development (“rapid” and “ballistics”), ending at two different levels of torque	Acute	Both RP and MP showed similarity with real and imaginary movements, independent of the strength development rate and torque amplitude.	FC1, FC2, CF13, CF1, CFZ1, CFZ2, CF2, CF24, C3, C13, C1, CZ1, CZ, CZ2, C2, C24, C4, CP3, CP1, CPZ1, CPZ, CPZ2, CP2 and CP4
Schillings et al. [9]	14 women	Flexors and extensors of right hand fingers	30 minutes of repetitive contractions with 70% of MVIC in handgrip with a 7 second interval between each grip	Acute	During repetitive contractions the beginning of RP changed from 1.5 second to 1.9 s before the strength start in Cz, and from 1.0 second to 1.6 and 1.7 second before the strength start in C3 and C4, respectively.	C3, Cz, C4
Falvo et al. [26]	9 women and 2 men	Vastus lateralis muscle of the quadriceps	3 times per week (total of 9 sessions); sessions 1 and 3–3 x 10–12 repetitions with 70-75% of 1RM; sessions 6 and 4–4 x 8–10 repetitions with 75-80% of 1RM; sessions 9 and 7–5 x 6–8 repetitions with 80-85% of 1 RM	Chronic	Amplitude attenuation of MP in Cz, C1 and C2 (p < 0.05) in post-workout. RP was started in advance at 28% to the Cz electrode in the post-workout	C1, Cz, C2

*Abbreviations*: *MRCP* Movement-related Cortical Potential; *SMA* Supplementary motor area; *MVIC* Maximum voluntary isometric contraction; *MVC* Maximum voluntary contraction; *NS* Negative slope; *MP* Motor potential; *RP* Readiness potential; *RM* Maximum repetition; *SFC* Chronic fatigue syndrome; *FT* Task with fatigue; *NFT* Non-fatiguing task.

## Discussion

This review aims to provide precise and chronological information related to current research and the main conclusions related to MRCP behavior in counter-resistance training.

The results of the studies presented in this review indicate a correlation between MRCP and the following factors: strength levels [8,12], strength development rate [7,12], induced fatigue [9-11,22,23] and specific phase of muscular action [24,25]. However almost all these studies mentioned above show acute results. Only one was found focused on the MRCP behavior showing neural adaptations with counter-resistance training [26]. Two sections were inserted in the text discussion for further clarification on the acute and chronic effects of counter-resistance training on MRCP behavior.

### MRCP and acute effects of muscle strength

The selected studies addressed the MRCP behavior by some different factors, such as strength levels [8,12], strength development rate [7,12], induced fatigue [9-11,22,23] and specific phase of muscular action [24,25].

#### MRCP and strength level

MRCP seems to have good correlation with strength magnitude. The studies conducted by Oda and Moritani [8] and Siemionow *et al*. [12] demonstrate that the larger the magnitude of strength required for static movements, the greater is the extent of brain cortical signals originated from the supplementary motor area, the sensorimotor cortex and premotor cortex.

The research conducted by Oda and Moritani [8] demonstrates that, in the frontal electrode (Fz), the probability of negative slope (NS) with 50% of maximum voluntary isometric contraction (MVIC) was significantly higher than for 10% of the MVIC). In the central areas (C3 and C4), with 50% of MVIC tends to be greater than 10%, but significant differences were not observed. Nonetheless, the signals generated in C3 were significantly higher (p < 0.01) than in C4, indicating contralateral predominance of movement, since the right arm was used.

In the study conducted by Siemionow *et al*. [12] the relationship between MRCP with muscle voluntary activation was investigated. Four different strength levels were used in this study (10, 35, 60 and 85% of MVIC) for a static bending movement of the elbow, demonstrating that the higher the strength, the greater were the amplitudes of MRCP. Results of correlation analysis indicated that strong positive correlations between strength and MRCP were found at both sites of the cortex, supplementary motor area and sensorimotor. The authors concluded that MRCP may represent the cortical commands according to the strength level for muscle activation [12]. In addition it is appear that initial adaptations occur at upper levels of motor processing (supraspinal) before than spinal level. Thus perhaps be important observing MRCP for monitoring level of neural individual’s adaptations related to strength training.

#### MRCP and rate of strength development

The amplitude of each MRCP component depends on the number of involved neurons, the degree of synchronism and the discharge rate of these neurons during the period corresponding to MRCPcomponents [10]. Since muscle strength is directly related to the number of recruited spinal motoneurons and their temporal pattern, a stronger neural input is needed for a faster contraction in a given strength. The hypothesis that a higher rate of strength development is related to greater amplitude of MRCP has been investigated [12]. To verify this, an isometric contraction of elbow flexors at 30% of MVIC was performed at three different rates of strength development (low, intermediate and fast) [12]. MRCP was highly correlated with the rate of strength in both electrodes. The correlation between MRCP and the rate of strength was r = 0.84 (SMA) and r = 0.85 (sensorimotor area). The correlation between EMG and the rate of strength increase was high, with r = 0.85 for the brachial biceps muscle and r = 0.87 for the brachioradialis muscle. These results suggest a modulation of the strength development rate by MRCP [7]. In this study, the authors verified that in tasks involving imaginary movements of plantar flexion, the information related to MRCP seem to be encoded in the supplementary motor area (SMA) and in the “primary motor cortex” (M1) [7]. These MRCP consisted of readiness potential (RP) and motor potential (MP). A comparison between the imaginary and real movements demonstrated that the potentials related to the movement were similar, but they significantly differ in amplitude. One of the possible suggestions to explain this difference may be the withdrawal of ongoing motor programs.

Thus, the negative potentials that precede and co-occur with the movement are generated in specific areas and are highly correlated with the rate of strength development. If a rate strength development depends of cortical output signals, motor control should be emphasized in activities of force and power because technically better fast movements will be performed. It is valid not only in simple strength training but mainly in sports where athletes depend of precise motor control and powerful movements. To evaluate MRCP may be a plus in the training because simple details make the difference among better athletes.

#### MRCP and muscle fatigue

Following this line of investigation of MRCP in relation to muscle strength, some studies were carried out to investigate EEG patterns in the temporal domain with dynamic changes to a muscular performance as a function of muscle fatigue [27].

An increased muscle performance may be obtained by increasing the MRCP amplitude as a manner to compensate the peripheral muscle fatigue induced by exercise. This can be observed during repetitive contractions, where the area under the curve of the readiness potential almost doubled in the central motor cortex electrode (Cz) and increased four times to the electrodes of motor cortex located in the left (C3) and right (C4) hemisphere [9]. Even when the increase in readiness potential during repetitive contractions is clearly excessive, signals of peripheral fatigue were almost absent, since the median frequency of the amplitude of EMG signal was not significantly changed by fatigue in MVIC. However, it was demonstrated that fatiguing muscle contractions at 80% of MVIC are accompanied by an increase of RP.A reduction in RP was not observed during repetitive contraction of the hand at 50% of MVIC [22].

In an attempt to create a peripheral muscle fatigue state, Shibata, Oda and Moritani [10] verified the amplitudes of MRCP in different conditions of isometric contraction of elbow at 20% of MVIC and assessed the activity with and without arterial occlusion. The authors verified that under conditions of arterial occlusion the average of EMG amplitude in the output strength was significantly higher than without occlusion [10]. This increased amplitude of EMG may be due to progressive recruitment of additional motor units to compensate the deficit of strength during the task with arterial occlusion. The average amplitude of MRCP after appearance of movement was significantly higher with occlusion in all central electrodes (C3, Cz, C4). Therefore in conditions of necessity of more motor units cortical activation appears of great utility. In this condition MRCP may be increased by somatosensory inputs that inform to central nervous system the necessity of more peripheral activity for continuing contraction. Thus in tasks where is required continued effort, the coupling between peripheral and central nervous system appears be fundamental.

The MRCP of patients with chronic fatigue syndrome was compared with healthy subjects [11]. The results demonstrate that at 50% of MVIC the amplitude of MRCP for the combined tasks with and without fatigue was higher in patients than in the group of healthy subjects. Moreover, in the group with chronic fatigue syndrome, MRCP was greater for the job with fatigue than for the task without fatigue, whereas this was not observed for healthy subjects performing both tasks [11]. It seems that the level of peripheral fatigue may be responsible for such compensation, since the higher amplitude of MRCP in patients who already suffered from chronic fatigue was higher under fatigue conditions, compared to conditions without fatigue. However, it should be emphasized that fatigue induced by maximal voluntary contractions has differential effects on cortical signals during the preparation of motor tasks in relation to its implementation and maintenance (20). In this study, despite a strength decline in the execution of handgrip and the electromyographic signal of the finger flexors, the amplitude of negative slope (NS) did not change significantly in any of the five electrodes of EEG (C3, Cz, C4, Fz and Pz) due to muscle fatigue. Moreover, when repetitive movements at 20% of MVIC were performed requiring a high degree of concentration and attention to fit exactly to this small level of strength, an increase of RP also occurred, perhaps due to a greater intended participation [22]. It seems that the complexity of the motor task caused this greater response of MRCP, since the accomplishment of motor tasks discloses that the amplitude of MRCP is lower, since in motor tasks amplitude of MRCP is lower when making the same movement in a regular manner when compared with different (random) movements being regularly made [28]. MRCP was higher in random tasks, suggesting that the extra activities in sensorimotor areas reflect processes involved in motor preparation rather than memory and attention processes. Since the random task was very simple, the mechanisms of attention and memory were similar to regular tasks. The random tasks showed more activity than the regular task in contralateral frontal areas. Thus, it was demonstrated that the greater the complexity of motor task, the higher the MRCP [28].

#### MRCP and muscle actions

Although there is great evidence that there may be different strategies of the nervous system for controlling concentric and eccentric muscle actions, MRCP has been scarcely investigated as an useful tool to further elucidate these mechanisms. Most studies [8,12,29] have used static contractions.

Thus, as was previously observed, in static strength tasks, the greater the magnitude of the strength, the greater MRCP will be. However, comparisons of MRCP in different muscle actions have been investigated in concentric and eccentric actions. It was demonstrated that despite the higher electromyographic activity of elbow flexor muscles in concentric actions, the amplitude of negative slope (NS) of MRCP was significantly higher for eccentric action than for concentric action [25]. The appearance of MRCP for the eccentric task occurred earlier than for concentric tasks. The greatest cortical sign of eccentric muscle actions suggests that probably the motor planning and programming of the brain occur differently in eccentric muscle work for concentric muscle movements.

Another important point that should be emphasized is the strength magnitude in different muscle actions. Fang et al. [24] verified if the responses of MRCP were different in concentric and eccentric actions, and now they used contractions of maximum intensity, since in their previous study [25] contractions had submaximal intensity. The results demonstrated that although the activity at the level of flexor muscles in the elbow is lower in eccentric movements than in concentric movements, MRCP indicated a higher cortical activation, both in magnitude and dimension in the area for that eccentric task. A detailed comparison of each electrode signal suggested that a longer preparation time was needed, as well as a greater magnitude of cortical activity for eccentric movements to delay the movement execution. The analysis of electrodes C3, C4, C6, F4, FC4 and FC6 demonstrated that the amplitude of negative slope was greater in eccentric than in concentric actions, besides of a longer period of latency for activation.

The additional preparation time and higher amplitude of activation may reflect a different activity of CNS, since eccentric movements are associated with higher risk of injury. It suggests brain activity is more important for eccentric motor control than peripheral activity demonstrated by comparison between EEG and EMG. Time preparation and magnitude of signals in eccentric contractions show that CNS needs more time of planning and preparation for eccentric movement control. Perhaps CNS requires more time of adaptation for great eccentric control. This may be essential in athletes training (eg. sprinters and jumpers) that perform fast eccentric movements.

### MRCP and chronic effects of muscle strength

In this review, only one study was found which intended to investigate the chronic effects of counter-resistance training on MRCP [26].

The subjects performed unilateral explosive counter-resistance training for the leg extensor muscle during three weeks, three times a week. This training consisted of sessions as follows: sessions 1 to 3–3 × 10–12 repetitions with 70-75% of 1RM, sessions 4 to 6–4 × 8–10 repetitions with 75-80% of 1RM and sessions 7 to 9–5 × 6–8 with 80-85% of 1RM. Before and after three weeks of counter-resistance training 60 knee extensions against a fixed load were performed as strength evaluation. The results demonstrated increases in MVIC (21.6%), rate of strength development (31.6%) and average of EMG integrated signal over the time interval of 200 ms before the peak strength (47,2%). After training, the amplitude of MRCP was significantly attenuated in several overlying areas related to the areas of the motor cortex (Cz, C1 and C2) anticipated by 28% for the central electrode (Cz). Therefore, the three-week training protocol caused significant strength gains that were accompanied by neural adaptations at the cortex level. This study reinforce our previous idea where CNS can updating before peripheral structures and it is an important variable to measure motor performance related strength. Extrapolating this knowledge may be possible that persons with more adaptation of CNS can develop a better strength and motor control.

## Conclusion

The results of these adaptations in the motor cortex, i.e., neural adaptations, may be associated with increased amplitude of the MRCP by factors such as strength level, strength development rate, induced fatigue and specific stage of muscle action, favoring higher amplitude in eccentric actions compared with concentric actions. The long-term adaptations demonstrate that the counter-resistance training causes attenuation in amplitude in related areas, which may be due to neural adaptations that occurred at the motor cortex level. These findings suggest that in tasks of strength supraspinal neural network has an important role on performance of motor control and the long-term this activity decreases because these neural adaptations. Therefore individuals that present decreasing of motor cortex activity in strength tasks a long time may have better development of fast and strong movements.

## Competing interests

The authors have no conflict of interest or other sources of financial and material support related to this paper.

## Authors’ contribution

All Authors participated in the definition of the study design and the protocol. Authors EL, OAC, SM managed the literature searches. Authors EL, OAC, RSMJ, EMMP, SM, wrote the first draft of the manuscript. All authors contributed to and have approved the final manuscript.
